# Lifestyle, vulnerability to stress and prevailing health conditions of ambulatory older patients in a care facility

**DOI:** 10.4314/ahs.v23i1.58

**Published:** 2023-03

**Authors:** Uchenna IH Eze, Babatunde A Adeniji, Chinonyerem O Iheanacho

**Affiliations:** 1 Department of Clinical Pharmacy and Biopharmacy, Faculty of Pharmacy, Olabisi Onabanjo University, Sagamu, Ogun State, Nigeria; 2 Department of Clinical Pharmacy and Public Health, Faculty of Pharmacy, University of Calabar, Cross River State, Nigeria

**Keywords:** Lifestyle, older adults, vulnerability to stress, health conditions, health care facility, Nigeria

## Abstract

**Background:**

Lifestyle and vulnerability to stress are major determinants of age-related health outcomes.

**Objectives:**

To assess the lifestyle and health states of older adults, and evaluate their personality-related vulnerability to stress, to enable improved and targeted health promotional activities.

**Methods:**

A hospital record review and a purposive cross-sectional study was conducted among 200 respondents who were ≥ 50 years old, and visited the General Hospital Oyo, South-western Nigeria. Descriptive statistics was performed using SPSS version 21. Analysis of vulnerability to stress was performed by the addition of scores from Marshal's personality stress prone test. Lifestyle were measured by frequencies and Chi-Square tests, while presence of chronic diseases was measured by respondents' past prescriptions, from the hospital case notes. P < 0.05 was considered statistically significant.

**Results:**

A total of 200 respondents participated in the study. Majority, 156 (78.0%) were 50-59 years old and self-employed 96 (46.0%). Ninety-three (46.5%) smoked, 65 (32.3%) consumed alcohol, 128 (64.0%) had periodic exercise and 67 (33.3%) experienced insomnia. Majority (60.5%) were vulnerable to stress, and this was significantly associated with age (P=0.001), marital status (P=0.021), body weight (P=0.05), occupation (P=0.002) and income (P=0.002). From the retrospective study, most frequently prescribed drugs were anti-hypertensives 225 (69.7%), vitamins/minerals (49.5%), sedatives 158 (48.9%) and analgesics 158 (48.9%) respectively.

**Conclusion:**

Periodic exercise, alcohol use, and smoking were reported at varying degrees. High prevalence of vulnerability to stress and use of anti-hypertensives were also observed, and vulnerability to stress was associated with selected socio-demographics. These findings reinforce the need for routine education of this category of populace on healthy lifestyle for improved health.

## Introduction

Growing old is an important process that is widely gaining special attention as a result of its associated health and social issues 1. It is associated with several challenges and risk factors, among which are chronic diseases and psychosocial conditions. Ageing usually results in cellular and molecular damage, leading to progressive risks for diseases and death. Among other risk factors, age is considered to be best indicator of the risk of dying 2. Likewise, functional capacity progressively diminishes, thereby increasing susceptibility to health problems and consequently the possibility of death. The mortality rates among older patients increases for both sexes with age.

Lifestyle-related health effects are evident across all age groups but the impact of unhealthy living is different in older persons 1. Lifestyle related risks in the older population are most times, precursor to chronic illnesses and disabilities 3. Although, age-related metabolic changes occur in older persons 4, lifestyle associated risks of cardiovascular, metabolic and musculo-skeletal diseases in older adults are yet to be established 5. Other clinical conditions associated with older persons are risk of negative drug interactions and inappropriate dosage adjustment as a result of altered metabolic changes 4. Other conditions that may affect the wellbeing of older persons are psychological conditions resulting from loneliness, socioeconomic status, policies and environmental factors 6. Socioeconomic status is a common factor that influences both physical and mental health of ageing individuals 6.

Lack of access to health facilities and poverty are especially peculiar to older persons in low- and middle-income countries 6. These conditions which are usually chronic may result in vulnerability to stress in this group of persons^7^. The presence of stress, how individuals interpret such as stressful, the resources available to them and other intervening variables are predictors of chronic disease conditions in older persons. Available evidence also suggests that the presence of a chronic disease, the quality of support and available resources to an older person may significantly influence stress level and well-being 8,9,10. Stress arises from response to certain conditions which could be psychological or biological 11.

Vulnerability to stress in the older population may therefore be relative and lifestyle-related. It involves a tendency to have severe episodes of negative or distressing effects, or susceptibility to stress as associated with lifestyle. It could be measured using various tools such as tools for assessing stress prone personality 12, and may be associated with unhealthy lifestyle 11. Several socio-cultural attributes and practices commonly found in Nigeria are potential major contributing factors to stress and other risks in the older persons, and some of these are large number of dependents, especially the extended family, and low income, among others. The associated responsibilities tend to result in increased tension and anxiety among the older population 10. Older persons in Nigeria are also recognised to be faced with several psychosocial problems which influences their health seeking behaviour^13^. Common factors seen to influence psychosocial health of older persons in Nigeria include increased economic stress, decreased functional independence and increased demand for healthcare services 14. Meanwhile, there is scarcity of data on vulnerability to stress in Nigeria's older persons.

This article focused on vulnerability to stress and modifiable lifestyle that may influence risks for chronic diseases among older persons. Risk represents the possibility of occurrence of adverse effects and events 8. Occurrence of some essential and potential lifestyle risk factors which include smoking and alcohol use, lack of regular exercise, poor diet and poor sleep were also examined. Lifestyle and stress are important factors that may impact the health of older persons. Therefore, this study was aimed at assessing the lifestyles of older adults, their vulnerability to stress and the prevailing health conditions among them, to enable improved targeted health promotional activities.

## Methods

### Study setting and study design

The study was conducted in an out-patients' department of General Hospital Oyo, South-western Nigeria. Being a reference public hospital, it receives referrals from primary healthcare clinics, and provides healthcare to all categories of persons, including older persons. It is also a first point of call for most community residents who require medical attention, without referrals. Hence, it serves a large number of people within and outside the community. The study consisted of retrospective and purposive cross-sectional survey methods, and data were collected over a one-month period. The retrospective survey involved a review of relevant hospital records.

### Study population

The cross-sectional survey was conducted among 200 male respondents who were 50 years old and above. These were patients who visited the hospital within the past one month for various medical conditions, and sample size was calculated using Cochrane formula 15. Women are more likely than men to report symptoms of stress 16, and as such, stress may be under-reported in men. Therefore, this study is focused on understanding the vulnerability of older males to stress.

### Inclusion criteria

• Older patients who could understand and provide informed consent.

• Only older male out-patients were included in the study.

• Only out-patients who were 50 years old and above.

### Exclusion criteria

• Patients who did not provide informed consent.

### Data collection

The patients were recruited purposively and interviewed using a self-completion, structured questionnaire. Data collected included socio-demographic information, data on habits or lifestyle and vulnerability to stress. The medical records were also examined retrospectively to validate data provided by the patients, and to collect data on the patients' last five prescription drugs.

### Data collection tool

The questionnaire had three sections. Section A had nine (9) items on socio-demographic characteristics: Age, Weight, Religion, Occupation, Employment status, Monthly income, Present place of work, Marital status and Number of wives. Section B had 15 questions on habits and lifestyles: Smoking, alcohol intake, consumption of junks (fast foods), sleep time, wake-up time, recess, exercise and hobbies. Section C was adapted from previous research 12, and had six (6) questions with three (3) sub-options on vulnerability to stress (Personality stress test).

The questionnaire was validated through pretesting and face validity. The pretesting was done among randomly selected 15 older patients who visited the hospital 3 weeks prior to the study, after which modifications were made to remove ambiguity.

### Data analysis

The primary outcome measure was respondents' recreational lifestyle, while the secondary outcome measure was vulnerability to stress.

Obtained data were entered using Microsoft Excel, while IBM SPSS version 21 was used for the analysis. Descriptive statistics was done and the categorical variables were presented in percentages and frequencies. Further analysis was done using crosstabs and chi square. P-value at less than 0.05 was considered significant.

For the ‘Personality stress prone test’ section, vulnerability to stress was analysed by the addition of scores as follows: for every yes answer to an ‘a’, ‘b’ and ‘c’ questions; a score 6, 4, 2 were given respectively. A summation of the scores was made and the following interpretations were made for the following scores:

**a)**. 24 - 36= Living at a high stress pace and might be prone to coronary heart disease, Ulcers, and other stress related illness. (Counsel to receive - slow down, take time to relax. Look at your philosophy of life and perhaps take up a non-competitive hobby in your free time).

**b)**. 12-24- Relaxed and free from stress. (Counsel to receive - However, a certain amount of stress is healthy and a spur to positive achievements. Being less apathetic may help in having more achievements).

0-12- You create stress by inaction. (Counsel to receive - First try to relieve any symptom of stress, and then start a campaign to build up your confidence, self-esteem and assertiveness. Make a list of your good points and concentrate on them).

Although, there are few ways one can alter the way in which reaction to situations or people around them are expressed, it is difficult to change those aspects of personality that have been present from an early age, therefore changing them requires firmness of purpose. It is well worth persisting the attempts, especially if a personality presents health risks.

Vulnerability to stress was defined as personality-related attributes that predispose a person to stress. This was presented in a frequency table, and stress levels were presented in a bar chart.

Lifestyle was defined as activities that are routinely performed by a person, and these were presented in frequency tables. It was measured by frequencies and Chi-Square tests, while presence of chronic diseases was measured by respondents' past prescriptions from the hospital case notes.

### Ethical statement

Approval was obtained from the ethics committee of the hospital management board, with the reference number: SHO/R/004/19. Written informed consent was also obtained from the study participants prior to the study.

## Results

A total of 200 patients participated in the survey, showing a response rate of 100 %. Table 1 shows the socio-demographic characteristics of the respondents. The majority, 156 (78.0 %) of sample population belonged to the age group of 50 and 59 years, and majority 96 (46 %) were self-employed. Over half 56 (28.0 %) were civil servants, and 20 (10.0 %) were retired. Body weight of 61 to 70 kg was mostly observed in the patients 90 (45.0 %).

**Table 1:** Socio-demographics of ambulatory older patients in a Nigerian healthcare facility. (N = 200)

Variables	Frequency	Percentage
**Age (Years)**		
50 – 59	156	78.0
60 – 69	40	20.0
**Above 70**	4	2.0
**Body weight (Kg)**		
30 – 40	6	3.0
41 – 50	80	40.0
51 – 60	24	12.0
61 – 70	90	45.0
**Religion**		
Christianity	168	84.0
Islam	32	16.0
**Marital Status**		
Married	178	89.0
Separated/Divorced/Single	22	11.0
**Number of wives (n = 180)**		
One	147	73.5
Two	21	10.5
Three	8	4.0
Four	2	1.0
**Occupation**		
Civil Servant	56	28.0
Professional	18	9.0
Self-employed	92	46.0
Artisan	4	2.0
Retiree	20	10.0
Others	10	5.0

Table 2 shows the recreational lifestyle of the respondents. Ninety-three (46.5 %) of the respondents smoked and more than half 117 (58.5 %) consumed alcoholic drinks which was mostly taken weekly 76(38.0 %). The percentage of those that consumed fast foods/junk foods and pastries was quite high, with a total of 127 (63.5%) of the study population consuming this at various degrees. Only half 100 (50.0 %) of the respondents earned 70,000 Naira (183.47 US dollars) and above, monthly. Some of the respondents, 67 (33.5 %) experienced insomnia, meanwhile majority, 137 (68.5 %) went to bed beyond 10 pm, and 122 (61.0 %) woke up from 5 am. Majority 128 (64.0 %) engaged in periodic exercise. Other attributes of the respondents that influence their risks are shown on the Table.

**Table 2:** Recreational lifestyle of ambulatory older patients in a Nigerian healthcare facility. (N = 200)

Variables	Frequency (n)	Percentage (%)	Variables	Frequency (n)	Percentage (n)
**Smoking**			**Insomnia**		
Yes	93	46.5	Yes	67	33.5
No	107	53.5	No	130	65.0
**Quantity**			Don't know	3	1.5
1–4	89	44.5	**Bedtime (p.** **m)**		
Don't know	4	2.0	7.00	6	3.0
Not applicable	107	53.5	8.00	22	11.0
**Alcohol intake**			9.00	35	17.5
Yes	117	58.5	10.00	133	66.5
No	82	41.0	11.00 and above	4	2.0
Don't know	1	2.0	**Wake up (a.** **m)**		
Type of drink (n - 199)			3.00	19	9.5
Wine	15	7.5	4.00	59	29.5
Beer	76	38.0	5.00	91	45.5
Shnapps	25	12.5	6.00 and above	31	15.5
Palm wine	1	0.5	**Observe a nap**		
None	82	41.0	Yes	87	43.5
**Frequency of intake (n= 199)**			No	61	30.5
Daily	20	10.0	Don't Know	24	12.0
Weekly	76	38.0	Sparingly	28	14.0
Monthly	21	10.5	**Exercise**		
Not Applicable	82	41.0	Yes	119	59.5
**Junk foods/fast foods**			No	44	22.0
Yes	127	63.5	Don't know	28	14.0
No	41	20.5	Sparingly	9	4.5
Don't know	32	16.0	**Type of** **exercise**		
**Income Naira (US dollars)**			Aerobics	109	54.5
≤19,999 (52.42)	16	8.0	Non-aerobics	19	9.5
20,000–39,999 (52.43 – 104.85)	26	13.0	Not applicable	72	36.0
40,000–59,999 (104.85 – 157.27)	42	21.0	**Frequency of** **exercise**		
60,000–69,999 (157.27 – 183.48)	16	8.0	Daily	70	35.0
70,000 & above (≤183.49)	100	50.0	Weekly	33	16.5
**Frequency on income**			Monthly	16	8.0
Daily	24	11.9	Sparingly	9	4.5
Weekly	74	36.8	Not applicable	72	36.0
Monthly	12	6.0			
Yearly	76	37.8			
Not applicable (irregular)	14	7.5			

*381.50 Naira = 1 US dollar

**Figure 1:**
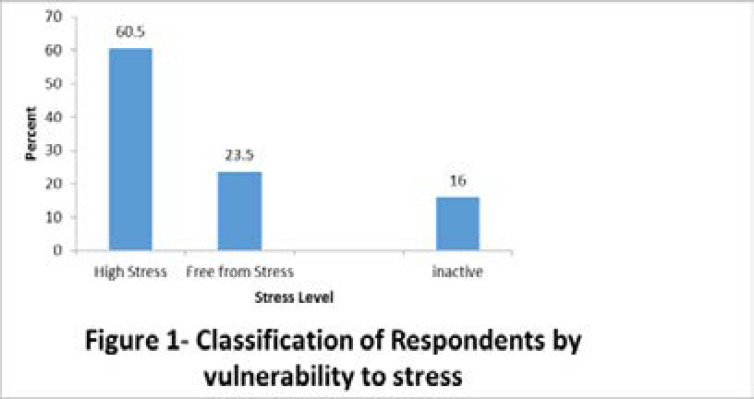
Classification of Respondents by vulnerability to stress

The patients' vulnerability to stress was described in Table 3 and Figure 1. Overall, majority of the respondents 132 (66.0 %) were very ambitious and competitive, 130 (65.0 %) were sacrificial, would not take “no” for an answer 129 (64.5 %). Meanwhile all the study participants 200 (100.0 %) reported to a habit of “performing all personal activities with speed”. The majority of patients were vulnerable to stress (60.5 %) and only 16.0 % were inactive. See Table 3 and Figure 1.

**Table 3:** Vulnerability to stress of ambulatory older patients in a Nigerian healthcare facility. (N = 200)

Question	YES		NO	
	
	N	%	N	%
1a) Are you competitive and aggressive at work sports and games	132	66.0	68	34.0
1b) If you lose a few points, do you sure up easily?	32	16.0	168	84.0
1c) Do you avoid confrontations?	124	62.0	76	38.0
2a) Are you ambitious and anxious to achieve a lot?	132	66.0	68	34.0
2b) Do you wait and expect things to happen to/for you	129	64.5	71	35.5
2c) Do you find excuses to put off things?	44	22.0	156	78.0
3a) Do you like to get things done quickly and often become impatient (Are you impatient?)	119	59.5	81	40.5
3b). Do you rely on other people to spur you into action. (Are you self-driving)	145	72.5	55	27.5
3c) Do you often rerun the events of the day and worry about them (Do you get worried?)	66	33.0	134	67.0
4a) Do you talk fast, loudly ad emphatically and interrupt a lot? (Are you forceful?)	47	23.5	153	76.5
4b) Do you take no for an answer with equanimity?	71	35.5	129	64.5
4c) Do you find it hard to express your feelings and anxieties (Are you articulate?)	159	79.5	41	20.5
5a) Do you get bored easily?	43	21.0	157	78.5
5b) Do you like having nothing to do. (Do you love Idleness?)	41	20.5	159	79.5
5c) Do you accommodate peoples feeling?	130	65.0	70	35.0
6a) Do you walk, eat and drink quickly? (Do you do things quickly?)	200	100		
6b) If you forget to do something, do you not bother (Do you feel okay when you forget things?)	54	27.0	146	73.0
6c) Do you bottle things up?	79	39.5	121	60.5

Table 4 shows association between respondents' socio-demographic variables and their vulnerability to stress. Age was statistically significant (P = 0.001) in respondents' vulnerability to stress. Other variables that were significantly associated with vulnerability to stress were body weight (P = 0.005), marital status (P = 0.021), occupation (P = 0.002) and income of (P = 0.002).

**Table 4:** Association between Respondents' Socio-demographic variables versus their vulnerability to stress

Variables	High Stress n (%)	Stress Free n (%)	Inactive n (%)	Total n (%)	Chi-square	P-value
**Age (Years)**						
50 – 59	89(44.5)	45(22.5)	22(11)	156(78)	X^2^ =21.976 df=6	**P=0 .001** *
60 – 69	30(15.0)	2(1%)	8(4.0)	40(20.0)		
70 – 79	0	0	2(1.0)	2(1.0)		
Above 80	2(1.0)	0	0	2(1.0)		
**Body weight (Kg)**						
30 – 40	0	4(2%)	2(1%)	6(3.0)	X^2^ =18.416; df=6	**P = 0.005** *
41 – 50	42(21.0)	24(12%)	14(7.0)	80(40.0)		
51 – 60	20(10.0)	2(1%)	2(1.0)	24(12.0)		
61 – 70	59(29.5)	17(8.5%)	14(7.0)	90(45.0)		
						P = 0.752
**Religion**						
**Christianity**	101(50.5)	41(20.5)	26(13.0 )	168(84.0)	X^2^ =0.571; df=2	
**Islam**	20(10.0)	6(3.0)	6(3.0)	32(16.0)		
**Marital Status**						
**Married**	108(54.0)	40(20.0)	30(15.0)	178(89)	X^2^ =14.847; df=6	**P =0 .021** *
**Separated**	1(0.5)	1(0.5)	0	2(1,0)		
**Divorced**	0	4(2.0)	2(1.0)	6(3.0)		
**Single**	12(6.0)	2(1.0)	0	14(7.0)		
**Occupation**						
**Civil Servant**	29(14.5)	15(7.5)	12(6.0)	56(28.0)	X^2^ =22.415; df=6	**P = 0.002** *
**Professional**	8(4.0)	6(3.0)	4(2.0)	18(9.0)		
**Self-employed**	64(32.0)	22(11.0)	6(3.0)	92(46.0)		
**Artisan**	2(1.0)	2(1.0)	0	4(2.0)		
**Retiree**	14(7.0)	0	6(3.0)	20(10.0)		
**Others**	4(2.0)	2(1.0)	4(2.0)	10(5.0)		
**Income Naira (US** **dollars)**						
**≤19,999 (52.42)**	8(4.0)	2(1.0)	6(3.0)	16(8.0)	X^2^ =24.068; df=8	**P = 0.002** *
**20,000–39,999**	20(10.0)	2(1.0)	4(2.0)	26(13.0)		
**(52.43. 104.85)**						
**40,000–59,999**	30(15.0)	4(2.0)	8(4.0)	42(21.0)		
**(104.85–157.27)**						
**60,000–69,999**	6(3.0)	6(3.0)	4(2.0)	16(8.0)		
**(157.27–183.48)**						
**70,000 & above**	57(28.5)	33(16.5)	10(5.0)	100(50.0)		
**(≤183.49)**						

* Statistically significant

**Figure 2:**
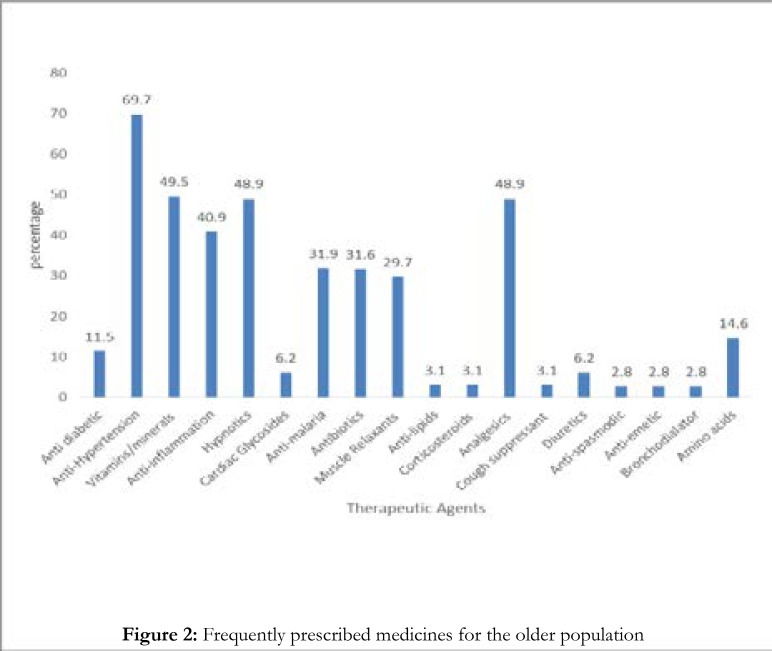
Frequently prescribed medicines for the older population

From the medical record review, the most frequently prescribed drugs for people of those age groups were the anti-hypertensive medications 225 (69.7 %), vitamins/minerals (49.5 %) analgesics 158 (48.9 %), sedatives 158 (48.9 %), anti-inflammation drugs 132 (40.9 %), and muscle relaxants 96 (29.7 %) respectively. Others are shown in Figure 2.

## Discussion

Among the several habits observed in the study participants were regular exercise, smoking, alcohol intake, consumption of junk foods and polygamy. Alcohol use was reported by a high percentage of the study participants, which is in contrast with a previous finding in Nigeria^17^, but consistent with findings from a previous study in India where high percentage of alcohol consumption was reported among the older population, with majority being heavy users 18. Older persons are very much susceptible to certain complications of heavy alcohol use such as Korsakoff syndrome 18,19. The risk in alcohol use in older persons is also dependent on a number of factors which include the frequency of use, high-risk behaviours, co-morbid conditions and concomitant use of other medications 20. Alcohol consumption has also been suggested to increase stress in the elderly 10.

Among participants who smoked, one to four sticks of tobacco at a time were most commonly reported. Smoking is a modifiable lifestyle that increases the risk of age-related co-morbidities. Age-related medical conditions also predispose the older population to harmful consequences of smoking 21. Similarly, frailty in old age has been suggested to be associated with smoking. This association which was demonstrated in a previous study showed current smoking to be a risk factor for quicker frailty development in older people 22.

The majority of respondents in this study had sources of income. The association of improved health and higher socioeconomic status has long been recognized 9, as money plays major role in everyday life, including in the social and psychological health of individuals. In a previous study, improved health status was observed with increase in income 9. It therefore plays a major role in the general wellbeing, particularly in this group of persons.

Insomnia was reported among the patients and prescription of sedatives was found in a high percentage of them. Over time, knowledge of age-related normal changes in sleep has grown, as reported by Cooke and Israel 23. Sleep disorders in older people may result from several medical conditions such as pain, cardiovascular and other chronic diseases, as well as medications used in treating these conditions^24^. The study showed that majority of the respondents had about seven hours of sleep. Although, several of them went to bed after ten p. m, majority also woke up after five a. m. This apparent moderate sleep duration may be associated with the use of sedatives as seen in the study. Several studies have shown that health outcomes such as morbidity and mortality could be predicted by sleep hours 22.

Healthy eating is an essential component of good health and hence cannot be over-stressed, especially for the ageing population. The majority of participants in this study ate junks/fast foods, which mostly comprise same class of food, usually carbohydrates. A diet that is composed of the six classes of food is said to be balanced, and gives a boost to the immunity, preventing both communicable and non-communicable diseases 25. This is particularly needed, considering the high burden of communicable diseases 26 and risks of non-communicable diseases in Nigeria 27. Nigeria is faced with a double burden of communicable and non-communicable diseases and as such conscious efforts are required towards ensuring healthy lifestyle. Effective educational strategies will be of high relevance in this regard.

Exercise is another major contributor to human health. Regular aerobic exercise has been linked to improved health in chronic conditions, including cardiovascular health 28. Participants in this study appeared to be familiar with the health consequences associated with exercise as more than half practiced aerobic exercise regularly. Daily exercise, as was reported by almost half of the study group is an indication of good understanding of the role of exercise in human health. Exercise is a type of physical activity that improves or maintains fitness 29. It aids in the prevention of non-communicable diseases^28^, with reduced risk and improved health associated with increased frequency and higher levels of exercise 30. It is recommended that exercise include aerobic exercise, strength exercise and balance exercise which reduces the risk of falls 29.

The findings show that majority of the respondents were vulnerable to stress as a result of their attitudes and approaches towards events, achievements, other people and activities. Stress and psychosocial factors are key determinants of several illnesses. Higher levels of perceived stress may result in higher mortality as reported in a previous study 31.

Vulnerability to stress was observed to be associated with age, marital status and low income. A previous study conducted in Iran, also reported low and middle income to be associated with high perceived stress 32. Socio-demographic characteristics are major determinants of health, and should normally be considered as priority in disease prevention approaches. Findings showed that marital status was associated with vulnerability to stress. Marital and family demands, may potentially raise the environmental effects of stress. Meanwhile, marital support and presence of a spouse reduces emotional reactions to stress, therefore married persons may report fewer symptoms of stress-related depressive symptoms 33. As a result of several biochemical changes and co-morbidities in older age, the risk of health-related impact of stress is of public health importance in this category of persons.

Also, findings from the retrospective study showed most frequently prescribed drugs for the group to be antihypertensive medications, vitamin/mineral supplements, sedatives and analgesics respectively. Hypertension may hence, be regarding as the prevailing health condition among the group. The observed predominant high level of vulnerability to stress among study participants may be responsible for the observed high prevalence of hypertension^34^. Vitamins and mineral supplements play a major in combating stress in persons of all ages and may be particularly useful in older person who are vulnerable to stress.

Although the study has shed light on the lifestyle and personality-related activities that may influence health in older persons, it is also faced with few limitations including, the self-reported process which poses a risk of self-reporting bias. Prevailing health conditions of the patients was assessed retrospectively by their last five prescribed medications, and not by use of definitive diagnosis. Also, this was a hospital-based study, which limits its generalization to the entire community.

## Conclusion

The study found several health-influencing habits in the study participants. Although at lower percentages, alcohol use and consumption of junk foods were found in a good number of the participants, while smoking was a habit in the majority. Regular exercise and presence of income were also seen to be predominant among them. Meanwhile, the participants were mostly vulnerable to stress by their personalities. Vulnerability to stress was also observed to be associated with selected socio-demographic characteristics. The hospital record review inferred prevailing medical conditions of these older population to include hypertension, insomnia and musculo-skeletal diseases respectively. These findings reinforce the need for routine education of this category of populace on healthy lifestyle for health promotion and disease prevention.
